# Natural Low-Eutectic Solvent Co-Culture-Assisted Whole-Cell Catalyzed Synthesis of Ethyl (*R*)-4-Chloro-3-Hydroxybutyrate

**DOI:** 10.3390/molecules30132869

**Published:** 2025-07-06

**Authors:** Yanni Wang, Bo Liu, Yanmei Dai, Zijuan Tao, Lan Tang, Zhimin Ou

**Affiliations:** 1 College of Pharmaceutical Science, Zhejiang Key Laboratory of Green Manufacturing Technology for Chemical Drugs, Zhejiang University of Technology, Hangzhou 310014, China; 13329103632@163.com (Y.W.); 211122070126@zjut.ecu.cn (Y.D.); 211122070059@zjut.edu.cn (Z.T.); tanglan@zjut.edu.cn (L.T.); 2 College of Biological & Environmental Sciences, Zhejiang Wanli University, Ningbo 315199, China; liubottbf666@163.com

**Keywords:** NADES, co-culture, cell permeability, whole-cell catalysis

## Abstract

In this study, CGMCC NO:28566, a strain that can efficiently convert Ethyl 4-chloroacetoacetate(COBE) to (*R*)-4-chloro-3-hydroxybutyrate((*R*)-CHBE), was screened by soil-sieving bacteria. In order to improve the transformation effect of the strain, the natural low-eutectic solvent (NADES), which can alter the cell permeability, was utilized for assisted catalysis, and a better catalytic effect was achieved. This study was carried out using a co-culture of strains with NADES and secondary addition of NADES on the basis of co-culture, and 10 NADESs were screened at the same time. The co-catalytic effect of 0.5% (*w*/*v*) choline chloride: urea (1:2) (ChCl:U (1:2)) was found to be the most significant, with a yield of (*R*)-CHBE reaching 89.1%, which was 58.2% higher than that of the control group, with a 99% ee value. Furthermore, the catalytic results demonstrated that the co-culture of the strain with NADES during fermentation yielded superior outcomes to the secondary addition of NADES during the reaction buffer. Furthermore, the catalytic effect of ChCl:U (1:2) was demonstrated to be superior to that of its individual components or single-component blends, due to its distinctive valence bonding advantage. The results indicate that the addition of 0.5% (*w*/*v*) ChCl:U (1:2) during the co-culture process has the effect of improving cell permeability to a certain extent, thereby increasing the contact between the substrate and the enzyme during the whole-cell catalytic reactions.

## 1. Introduction

Natural low-eutectic solvents (NADES) are composed of natural hydrogen-bond acceptors (HBAs) and natural hydrogen-bond donors (HBDs) [1,2,3]. The discovery of NADES offers a promising avenue for addressing the limitations of ionic liquids [4]. NADES can be used for extraction, solubility enhancement, and stabilization of bioactives [5]. In terms of composition, the natural hydrogen bond acceptors in NADES are predominantly quaternary ammonium salts, including betaine, dihydrocholine citrate, and choline chloride. In contrast, the hydrogen bond donors are primarily alcohols, sugars, organic acids, and urea [6].

The majority of pro-chiral ketone substrates are organic compounds that are insoluble in water. In order to facilitate the dissolution of the substrate, organic solvents such as isopropanol, glycerol, and ethanol are often employed to enhance the dissolution process [7]. Nevertheless, the addition of a considerable number of organic solvents can potentially cause damage to the cells. Consequently, researchers have focused their attention on the NADES, which are less toxic and have superior solubility properties [4]. Furthermore, NADES pre-treatment can enhance catalytic efficiency to a certain extent by modifying cell permeability, thereby increasing enzyme-substrate contact [8,9]. The advantages of NADES-assisted catalysis include NADES media ensuring higher activity for a wide range of enzymes, increased substrate solubility, and altered cell membrane permeability [10].

Isolation and purification of enzymes is a time-consuming process that will also result in the loss of enzyme content and the reduction in enzyme activity during purification, thus leading to a higher production cost [11]. In contrast, whole-cell catalysis enables the catalysis of the substrate without the destruction of the cells, which reduces the production cost and simplifies the production process [12]. Nevertheless, the principal obstacle to whole-cell catalysis is the challenge of mass transfer between intracellular enzymes and substrates in solvent systems [13]. The cell membrane of microorganisms exhibits a greater degree of selectivity in regulating the permeability of substances entering and exiting the cell [14]. In contrast, NADES can achieve changes in catalytic efficiency by altering the permeability of cell membranes [15]. The novel approach of co-culture with NADES represents a new type of assisted catalysis that pre-treats the permeability of bacterial cells during the enrichment stage of the bacterium. The method described by Qian et al. resulted in a notable enhancement in the production of chiral alcohols [9].

Ethyl 4-chloroacetoacetate (COBE) can be reduced asymmetrically to the enantiomers (*R*)-CHBE and (*S*)-CHBE [16]. (*R*)-CHBE can be employed as a pivotal intermediate in the synthesis of numerous pharmaceuticals. For instance, (*R*)-CHBE is utilized in the synthesis of antibiotics, macrolides, γ-amino-hydroxybutyric acid, cyclohexanone, and other compounds. Furthermore, it is employed in the synthesis of a plethora of pharmaceuticals, including antibacterial and anti-inflammatory agents, treatments for Alzheimer’s disease, cardiovascular disorders, and agents that address the dietary fatigue link and other related conditions [17,18].

In this study, the CGMCC NO:28566 strain, which is capable of efficiently converting COBE to (*R*)-CHBE, was screened by soil-sieving bacteria. Ten NADESs were then used to assist the catalysis of the CGMCC NO:28566 strain. The yields and ee% of (*R*)-CHBE obtained by catalysis of COBE by whole-cell strains were employed as reference standards, from which NADES that could effectively enhance the catalytic effect were identified. In parallel, the quantity of the preferred NADES added, the method of addition, the individual components and blends, as well as the pivotal reaction conditions, and the cell membrane permeability were subjected to further investigation.

## 2. Results and Discussion

### 2.1. Identification of the Best Strains

**Figure 1:**
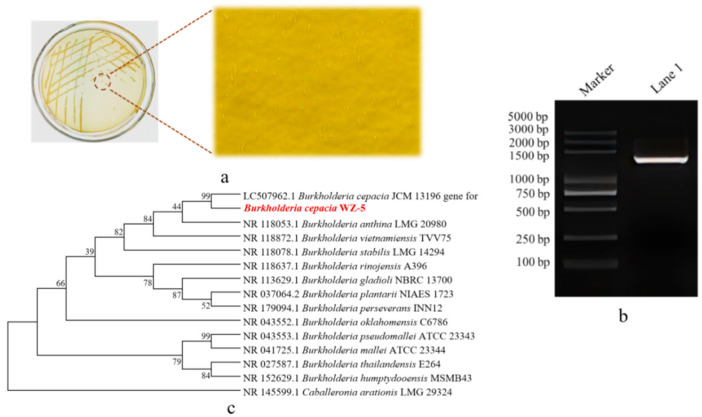
Identification of the best strains. (**a**) Microscope picture of strain No:28566; (**b**) Agarose nucleic acid electrophoresis of 16S rDNA sequences; (**c**) Evolutionary tree of the species.

The preferred strain, yielding (*R*)-CHBE at 95.7% and ee at 99%, was finally screened and named *Burkholderia cepacia* WZ-5. *Burkholderia cepacia* WZ-5 was preserved in the China General Microbiological Culture Collection Center and designated CGMCC No:28566. Microscopic observation, agarose nucleic acid electrophoresis of 16S rDNA sequences, and the evolutionary tree of the species are shown in Figure 1.

### 2.2. Substrate Conversion Analysis

**Figure 2:**
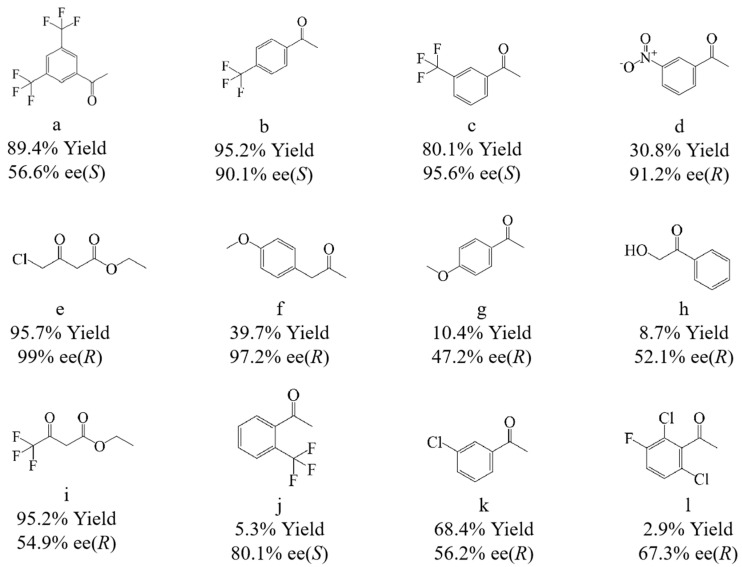
CGMCC NO:28566 strain catalytic substrate analysis. Reaction conditions: 10 mL PB buffer (pH 7.0), 120 g/L wet cells, 100 g/L glucose as co-substrate, 15 mM COBE, 200 rpm, and 25 °C for 30 h.

The substrate profile used by the CGMCC NO:28566 strain to reduce prochiral ketones was analyzed. As can be seen from Figure 2, the CGMCC NO:28566 strain has the ability to transform a variety of substrates, including a, b, c, and j, which are fluorinated aromatic ketones, and b has a 95.2% yield and 90.1% ee value. In addition, the strain also converted ester substrates such as e and i, and had a 95.7% yield and 99% ee value for COBE. COBE will be chosen as a substrate for further studies.

### 2.3. Effect of Medium Type on the Catalytic Activity of CGMCC NO:28566 Strain

**Figure 3:**
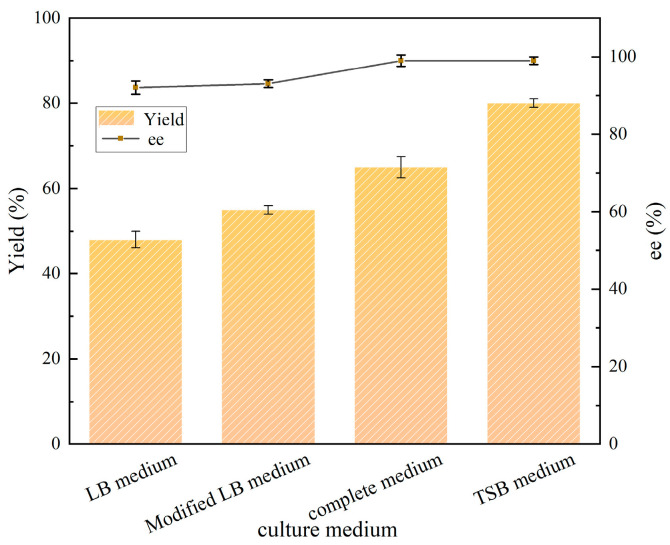
Effect of medium type on the catalytic activity of CGMCC NO:28566. Reaction conditions: 5 mL PB buffer (pH 7.0), 100 g/L wet cells, 100 g/L glucose as co-substrate, 15 mM COBE, 200 rpm, and 25 °C for 30 h.

As shown in Figure 3, LB medium, modified LB medium, complete medium, and TSB medium were selected as the basis for screening. Under the same reaction conditions, the yields of (*R*)-CHBE were TSB medium > complete medium > modified LB medium or LB medium, respectively, and the TSB medium had an 85.7% yield and a 99% ee value. It was postulated that the TSB medium was rich in carbon and nitrogen sources, as well as inorganic salts, which allowed the CGMCC NO:28566 strain to grow rapidly and demonstrate enhanced transformation effects. Consequently, TSB liquid medium was selected as the seed medium and fermentation medium for the CGMCC NO:28566 strain.

### 2.4. Effect of NADESs and the Method of Addition of NADES on the Reduction Activity of CGMCC NO:28566

**Figure 4:**
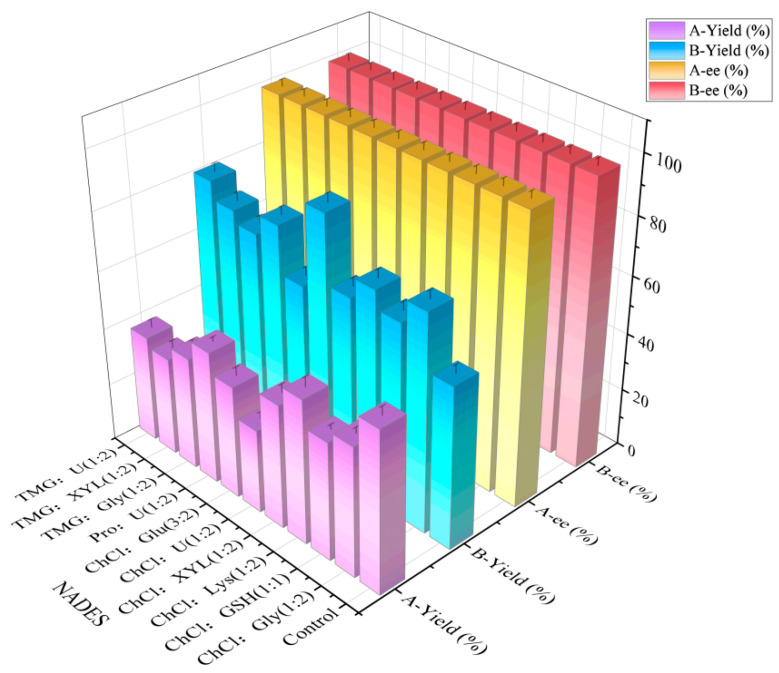
Effect of two NADES addition methods on reduction reaction. A: re-addition of 0.5% (*w*/*v*) ChCl:U (1:2) to the reaction buffer under co-culture conditions. B: co-culture treatment with 0.5% (*w*/*v*) ChCl:U (1:2).

As illustrated in Figure 4, all 10 NADESs (Table 1) exhibited enhanced activity compared to the control. For instance, betaine:urea (1:2), proline:urea (1:2), choline chloride:glycerol (1:2), and choline chloride:urea (1:2) were observed to facilitate catalysis, resulting in 42.8%, 37.5%, and 33.9% higher yields of (*R*)-CHBE compared to the control, respectively. The assisted catalysis of ChCl:U (1:2) had the most significant effect, with the yield of (*R*)-CHBE reaching 89.1%, which was 58.2% higher than that of the control group. Conversely, the co-culture-based secondary addition of NADES resulted in lower yields than the control group in all experimental groups, with the lowest (*R*)-CHBE yield reaching 29.6%. It was observed that, following the addition of two NADES solutions, the yield of the experimental group in which ChCl:U (1:2) was employed was found to be significantly lower. In this study, two methods of adding NADES were employed for reduction. The first method was co-culture treatment with 0.5% (*w*/*v*) ChCl:U (1:2). The second method involved the re-addition of 0.5% (*w*/*v*) ChCl:U (1:2) to the reaction buffer under co-culture conditions. The results indicated no significant differences in ee values between the two NADES addition methods. However, co-culture supplementation significantly enhanced (*R*)-CHBE yield compared to buffer re-addition.

When choline chloride is combined with urea in a 1:2 molar ratio to create a low-eutectic solvent, the resulting fluidic liquid contains anionic, cationic, and neutral molecules [15]. Upon the introduction of ChCl:U (1:2) into an aqueous solution, the presence of water molecules results in a disruption of the solvent’s structural integrity. This may result in the partial breaking of hydrogen bonds and the partial dissociation of the two components, choline chloride and urea [19]. In contrast, CGMCC NO:28566 was a Gram-negative bacterium. The cytoplasmic membrane of this bacterium contained a substantial quantity of peptidoglycan outside the membrane, which was employed to maintain the structural stability of the cell wall. The partially dissociated choline chloride cation is capable of interacting with the polysaccharide backbone and peptide chains in peptidoglycan, a process that is facilitated by hydrogen bonding or electrostatic forces [20]. It is also possible that Cl^−^ may interact with the cell membrane in a manner that could result in alterations to the cell wall or cell membrane structure [20]. The alteration may be slight or drastic, but the addition of urea as a hydrogen bond donor at this time alters or reduces the damaging effect. This can be explained by the fact that the addition of urea forms a hydrogen bond with the salt anion [21]. This mechanism reduces the toxicity of choline chloride to the cell while concomitantly modifying the structural and functional permeability of the cell wall and cell membrane [20]. The series of interactions described above results in a more stable binding of the substrate COBE to the enzyme, thus facilitating the completion of the catalytic process.

The results demonstrated that the solvent ChCl:U (1:2) exerted a more pronounced effect on the bacterial structure, yet caused greater damage when reintroduced during the substrate transformation phase [22]. It was therefore concluded that it would be more beneficial to assist the process of catalysis by affecting the bacterial structure during the growth period of the strain.

### 2.5. Effect of ChCl:U (1:2) Content on the Reduction Reaction

**Figure 5:**
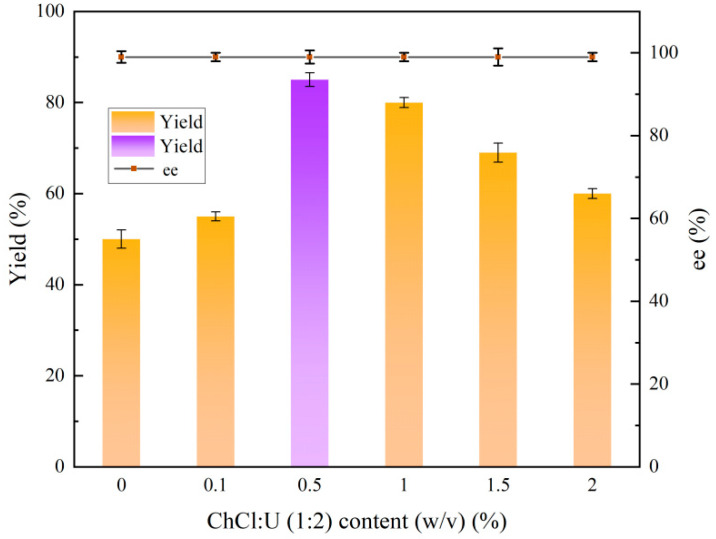
Effect of ChCl:U (1:2) content on the reduction reaction.

The results of the addition analysis showed that the concentration of ChCl:U (1:2) solvent required further optimization. Figure 5 illustrates that the addition of ChCl:U (1:2) had no significant effect on the ee value of (*R*)-CHBE. The results indicated that the addition of 0.1% (*w*/*v*) did not have a significant effect on the co-catalysis, with the yield of (*R*)-CHBE increasing from 50.9% to 53.6%. However, the (*R*)-CHBE yield increased from 53.6% to 85.4% at 0.5% (*w*/*v*) ChCl:U (1:2), indicating that this additive concentration significantly affected cellular structure. The yield of (*R*)-CHBE exhibited a notable decline at additive dosages between 1% (*w*/*v*) and 2% (*w*/*v*). This further demonstrates the low toxicity of the low-eutectic solvent as well as the investigability of the addition method. Consequently, adding 0.5% (*w*/*v*) ChCl:U (1:2) was more beneficial for enhancing (*R*)-CHBE yield in the strain’s fermentation culture.

### 2.6. Comparison of Optimization of Key Reaction Conditions Before and After Co-Culture Treatment

#### 2.6.1. Effect of Temperature on Reduction Reaction Before and After Co-Culture Treatment

**Figure 6:**
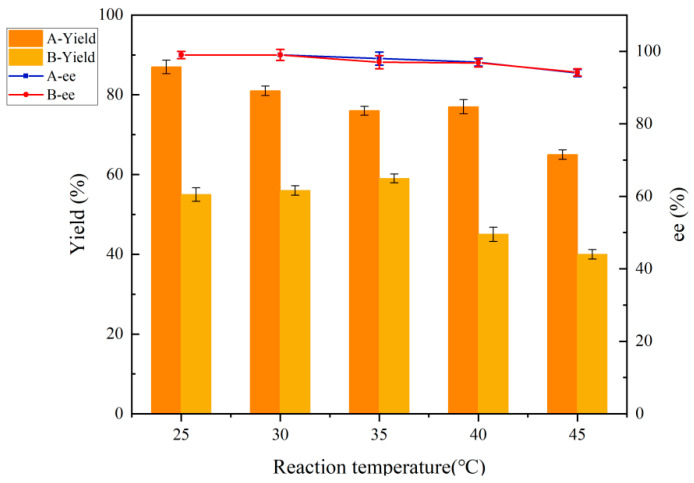
Effect of temperature on reduction reaction before and after co-culture treatment. A: co-culture treatment with 0.5% (*w*/*v*) ChCl:U (1:2). B: no co-culture treatment.

As illustrated in Figure 6, the yield of (*R*)-CHBE exhibited a notable reduction from 85.9% to 76.4% when the reaction temperature was elevated from 25 °C to 30 °C following co-culture treatment. Further increases in temperature, up to 35 °C and 45 °C, resulted in a yield of only 65.7%. This indicates that as the temperature rises, the activity of the cells, which are already partially altered in their cellular structure, is destroyed by the higher temperature. Nevertheless, the observed decline in ee value at temperatures between 40 and 45 °C may be attributed to a reduction in enzyme activity and stereoselectivity. Prior to and following the co-culture treatment, there was no significant decrease in yield, indicating that the CGMCC NO:28566 strain exhibits enhanced high-temperature resistance. The optimal reaction temperature for CGMCC NO:28566 in the absence of the co-culture treatment was 35 °C. In contrast, the co-culture treatment resulted in a reduction in the optimum selection temperature to 25 °C.

#### 2.6.2. Effect of Reaction Time on Reduction Reaction Before and After Co-Culture Treatment

**Figure 7:**
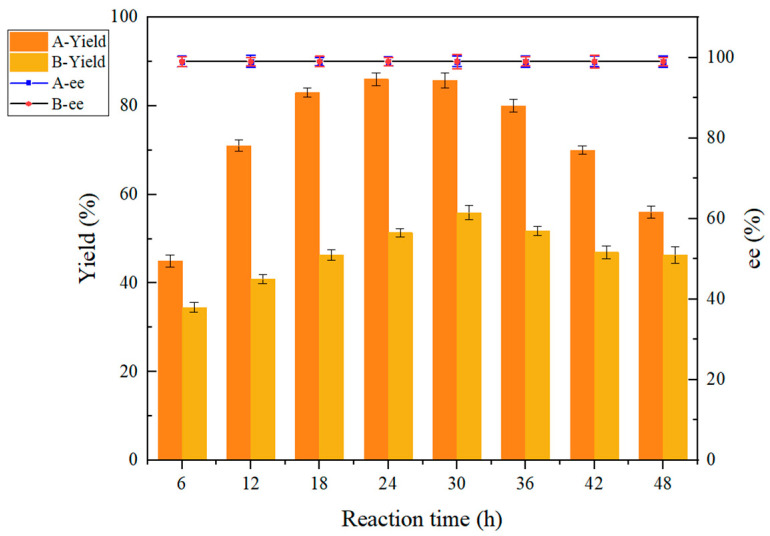
Effect of reaction time on reduction reaction before and after co-culture treatment. A: co-culture treatment with 0.5% (*w*/*v*) ChCl:U (1:2). B: no co-culture treatment.

The results of the analysis in Figure 7 demonstrate that the co-culture treatment had no significant effect on the ee value. The yield of (*R*)-CHBE exhibited a rapid increase, reaching 85.7% within the range of 6 h–24 h. Upon extending the reaction time to 30 h, the yield remained largely unaltered under co-culture conditions. At this time, the reaction time was extended to 48 h, and the yields of (*R*)-CHBE exhibited a decline to varying degrees. It is postulated that the observed decline in yield can be attributed to the accumulation of conversion products as the reaction time increases, a reduction in the conversion rate, and potentially other by-products. The optimal reaction time of CGMCC NO:28566 without co-culture treatment was 30 h, while co-culture treatment reduced the optimal reaction time to 24 h. Co-culture treatments increase yields while saving time.

#### 2.6.3. Effect of pH on Reduction Reaction Before and After Co-Culture Treatment

**Figure 8:**
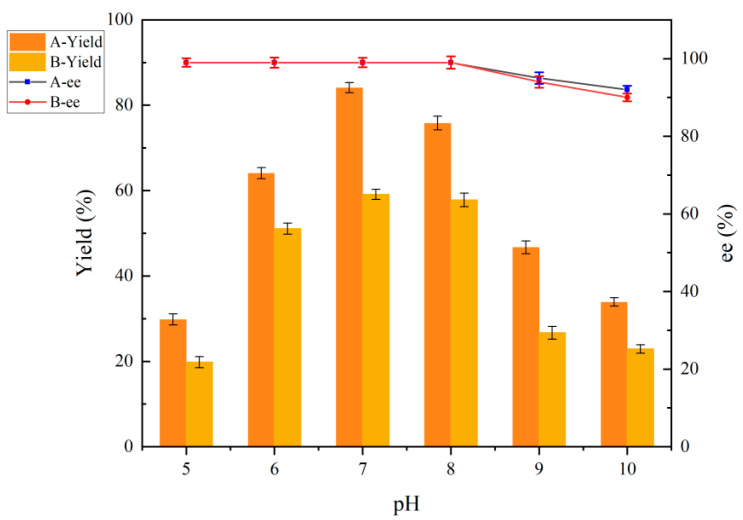
Effect of pH on reduction reaction before and after co-culture treatment. A: co-culture treatment with 0.5% (*w*/*v*) ChCl:U (1:2). B: no co-culture treatment.

The alteration in the cell structure under co-culture treatment conditions also affects the pH tolerance of the cells. As illustrated in Figure 8, the yields were found to be lower under more acidic conditions (pH 5.0–6.0) and more alkaline conditions (pH 9.0–10.0). The enzymes that play a significant role in this process were also subjected to more acidic or alkaline conditions, where enzyme activity was affected, and whole-cell catalytic efficiency was affected. The reaction was more favorable under neutral conditions, resulting in a yield of 86.7%. The optimal pH conditions were 7.0 before and after co-culture treatments.

### 2.7. Effect of ChCl:U (1:2) and Its Individual Components on the Reduction Reaction

**Figure 9:**
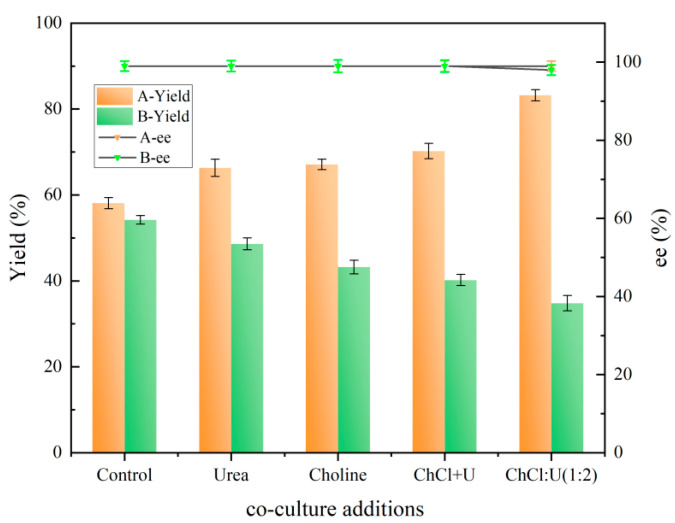
Effect of ChCl:U (1:2) and its individual components on the reduction reaction A: co-culture treatment with 0.5% (*w*/*v*) ChCl:U (1:2). B: re-addition of 0.5% (*w*/*v*) ChCl:U (1:2) to the reaction buffer under co-culture conditions.

The results of the analyses presented in Figure 9 indicate that neither the additional components nor the method of addition had a significant effect on the ee value of (*R*)-CHBE. The co-culture treatments of urea, choline chloride, or a simple co-mixture of choline chloride and urea resulted in increased yields of (*R*)-CHBE, with values of 66.3%, 71.2% and 71.2%, respectively. It can be analyzed that the co-culture treatments of urea, choline chloride, and a simple co-mixture of both did somehow increase the conversion capacity of the cells, but this effect is less obvious. However, this advantage is only apparent in the formation of ChCl:U (1:2), where the yield of (*R*)-CHBE increases from 58.1% to 83.2%.

However, the results from the repeated addition of choline chloride, urea, the simple two-component mixture, and ChCl:U (1:2) to the reaction buffer showed different decreases in (*R*)-CHBE yields. In addition, the effect of the repeated addition of ChCl:U (1:2) was also more pronounced in the above components, reducing the yield of (*R*)-CHBE to 34.8% compared to the control yield of 54.2%. It has been shown that low-eutectic solvents with choline chloride have some degree of biotoxicity [19], but that this toxicity may vary depending on the cellular system. The study also suggested that low-eutectic solvents based on choline chloride and urea are more biotoxic than either component alone or simple mixtures of the two, due to the high likelihood of ammonia production during the use phase of ChCl:U (1:2) [22].

### 2.8. Effect of ChCl:U (1:2) and Its Single Component on Cell Membrane Permeability

The process of improving catalytic efficiency by the degree of solvent action on the cell membrane, the components of the ChCl:U (1:2) solvent may also have a similar effect on the cell membrane. Typically, OD260 indicates the cellular nucleic acid leakage value, and OD280 indicates the cellular protein leakage value [23], so this subsection indirectly examines the change in cellular membrane permeability by testing the OD260 and OD280 indicators.

As illustrated in Table 2, the OD260 or OD280 values observed following 24 h of co-culture were found to be lower than those observed following 24 h of re-addition of the components presented in the table. In the case of secondary addition, the cell membrane structure may be more affected. The effect was significantly higher for ChCl:U (1:2) than for the single component and simple mixtures. Furthermore, the results of the assay after secondary addition of ChCl:U (1:2) to the reaction buffer reinforced this conclusion. Moreover, the measured OD260 or OD280 values corroborate the catalytic effect of Section 2.7.

### 2.9. FTIR Analysis of ChCl:U (1:2) and Its Single Components

**Figure 10:**
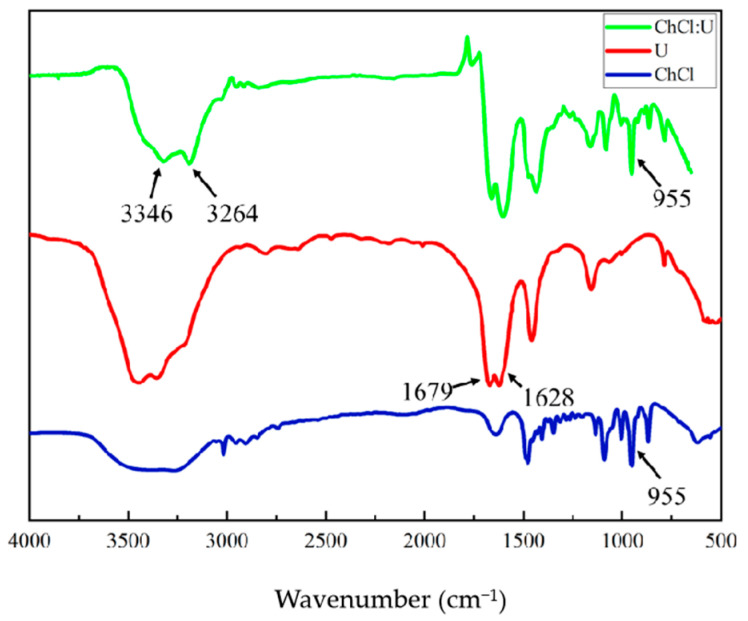
Infrared spectra of ChCl:U (1:2) and its single components.

From the infrared stacking diagrams in Figure 10, it can be observed that the infrared spectra of the ChCl:U (1:2) at the wavelength range of 4000–500 cm^−1^ combine the infrared spectral features of the choline chloride and urea. On the surface, the map appears to be a superposition of the maps of both choline chloride and urea. In comparison to the telescopic vibration absorption peaks of NH2 in urea at the wavelengths of 3346 cm^−1^ and 3264 cm^−1^, the absorption bands of ChCl:U (1:2) at this wavelength exhibit a broadening. Concurrently, the bending vibration of NH2 results in a shift of the absorption bands at the wavelengths of 1679 cm^−1^ and 1628 cm^−1^ towards the wavelengths of 1665 cm^−1^ and 1620 cm^−1^, respectively. The observed formation of hydrogen bonding between choline chloride and urea is indicative of the absorption peak at 1474 cm^−1^, which is attributed to the wobbling vibration of the CH3 group in the choline chloride molecule. In contrast, the absorption peak formed by the stretching vibration of the CCO group at 955 cm^−1^ in the spectrum of choline chloride remained after the formation of ChCl:U (1:2) low-eutectic solvent, indicating that the structure of Ch+ was not destroyed in the ChCl:U (1:2) solvent system.

Furthermore, the substantial number of hydrogen bonds that are formed during the preparation of this system indicates excellent flexibility properties [24]. The assertion that the interaction of choline chloride and urea forms hydrogen bonds during the formation of ChCl:U (1:2) is still the subject of some controversy. Nevertheless, the final conclusions indicate that this low-eutectic solvent possesses distinctive advantages over choline chloride or urea.

### 2.10. FCM Analysis Under Different Treatment Conditions of ChCl:U (1:2)

**Figure 11:**
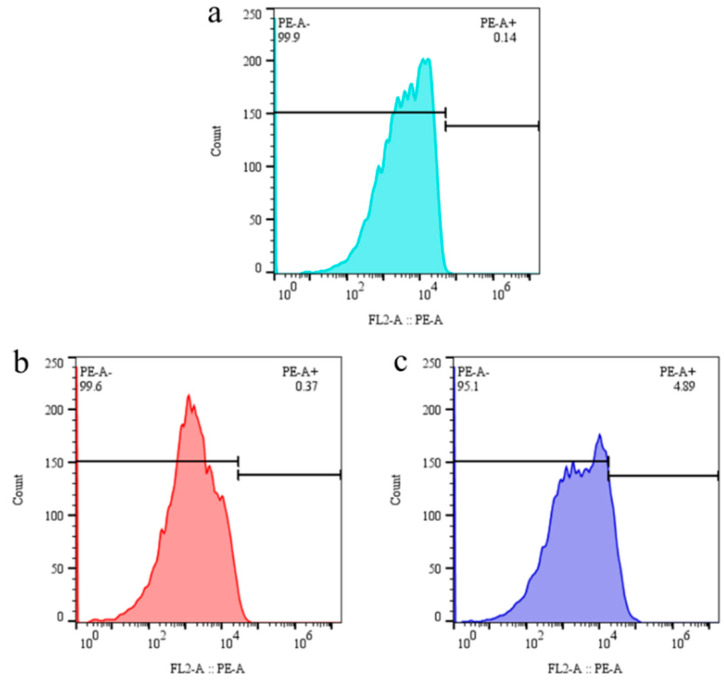
FCM analysis under different treatment conditions with ChCl:U (1:2). (**a**) Cells without co-culture treatment; (**b**) co-culture treatment with 0.5% (*w*/*v*) ChCl:U (1:2) for 24 h; (**c**) re-addition of 0.5% (*w*/*v*) ChCl:U (1:2) for 24 h in reaction buffer.

Propidium iodide (PI) [25,26] staining is a technique for staining cellular membranes that cannot penetrate the cell membrane of living cells. However, it can penetrate the cell membrane of broken cells, enabling nuclear staining [27]. The device exhibited a detection flux of 10,000 cell counts. The results of the flow cytometry (FCM) analysis, as presented in Figure 11, demonstrate that the cells treated with the three different approaches exhibited distinct outcomes. In particular, the 24 h co-culture treatment (PE-A+ 0.14) exhibited a notable difference in comparison to the control (PE-A+ 0.37). It can be surmised that during the period of co-cultivation with ChCl:U (1:2), the degree of alteration to the cell membrane is small, which confirms that the higher assisted catalysis results under this condition are based on lower damage to the cells. Furthermore, the result (PE-A+ 4.89) with ChCl:U (1:2) re-addition for 24 h on the basis of the co-culture (Figure 11c) appeared to be more pronounced than the control, indicating that this treatment condition caused greater damage to the cells. Consequently, the results demonstrate that the assisted catalysis effect was significantly diminished under the specified treatment conditions. The findings were in alignment with the outcomes of the OD260 and OD280 tests presented in Section 2.8.

### 2.11. SEM Analysis of CGMCC NO:28566 Strain Cells Under Different Treatment Conditions of ChCl:U (1:2)

**Figure 12:**
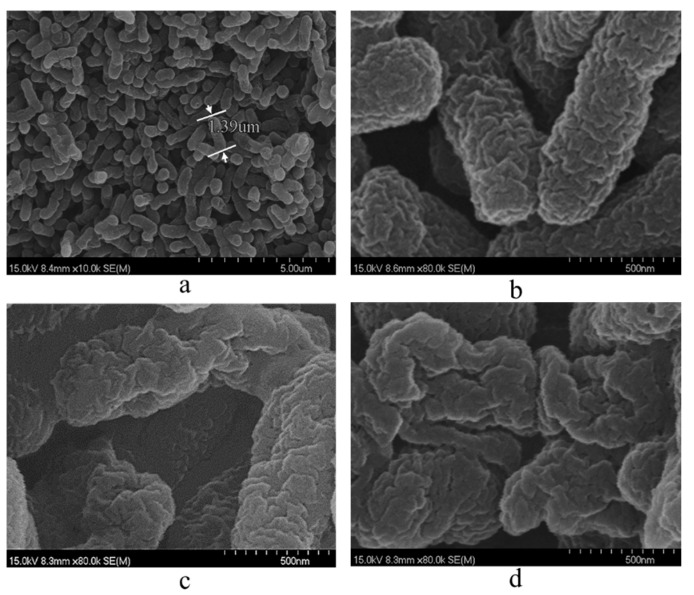
SEM analysis of CGMCC NO:28566 cells under different processing conditions with ChCl:U (1:2). (**a**) Appearance of CGMCC NO:28566 cells. (**b**) Cells without co-culture treatment. (**c**) Co-culture treatment with 0.5% (*w*/*v*) ChCl:U (1:2) for 24 h. (**d**) Re-addition of 0.5% (*w*/*v*) ChCl:U (1:2) for 24 h in reaction buffer.

Scanning electron microscopy was conducted on CGMCC NO:28566 cells following a series of treatment conditions involving ChCl:U (1:2) (voltage settings were 15 kV, magnification of Figure 12a was 10,000, and the remainder of the magnification was 80,000). As illustrated in Figure 12a, the electron microscope images of the CGMCC NO:28566 strain revealed an overall rod-like (or stick-shaped) morphology, which was consistent with the observations made using light microscopy (Figure 1). The length of the cells was approximately 1.39 μm. In comparison to the results obtained in the absence of ChCl:U (1:2) (Figure 12a), the surface of the cell exhibited varying degrees of alteration. Cellular depressions were observed in cells co-cultured with ChCl:U (1:2) for 24 h (Figure 12c). In contrast, cells that had been re-added ChCl:U (1:2) in reaction buffer for 24 h demonstrated an increased area of cell breakage and deformation (Figure 12d). Consequently, this also elucidates the catalytic outcomes delineated above, as well as the varying degrees of elevation of OD260 and OD280.

## 3. Materials and Methods

### 3.1. Materials and Reagents

The strains employed in the experiment were derived from a soil screening process. All NADESs (Table 1) were supplied by Shanghai Chengjie Chemical Co., Ltd. (Shanghai, China). Media-related reagents were obtained from Sinopharm Chemical Reagent Co., Ltd. (Ningbo, China). COBE (>98% purity, HPLC) and (*R*)-CHBE (>99% purity, HPLC) were procured from Rinn Technology Development Co., Ltd. (Shanghai, China).

### 3.2. Screening, Identification, and Culture of Microorganisms

The following culture media are relevant: enrichment liquid medium, glucose 25 g/L, yeast extract 3 g/L, ammonium sulphate 5 g/L, magnesium sulphate 0.25 g/L, dipotassium hydrogen phosphate trihydrate 1.5 g/L, and potassium dihydrogen phosphate 1.5 g/L. The pH was adjusted to approximately 7.0 with a 1 M sodium hydroxide solution. The restriction liquid medium comprised ammonium sulphate 5 g/L, magnesium sulphate 0.25 g/L, dipotassium hydrogen phosphate trihydrate 1.5 g/L, potassium dihydrogen phosphate 5 g/L, 10 mM COBE, deionized water as a solvent, and a final pH adjustment to 7.0 with 1 M sodium hydroxide solution. The solid medium was restricted, containing 5 g/L ammonium sulphate and 0.25 g/L magnesium sulphate. The medium consisted of 25 g/L dipotassium hydrogen phosphate trihydrate, 1.5 g/L potassium dihydrogen phosphate, 1.5 g/L potassium hydrogen phosphate, 10 mM COBE, 16 g/L agar, and deionized water as a solvent. The pH was finally adjusted to approximately 7.0 with 1 M sodium hydroxide solution.

The soil samples were collected from the provinces of Zhejiang, Jiangsu, and Shandong in China. The soil sieving procedure was as follows: approximately 0.5 g of each soil sample was dissolved in 5 mL of 0.9% saline solution, vortexed and shaken thoroughly for approximately 2–3 min, and then left to stand for 30 min. Two milliliters of the supernatant was then transferred to the enrichment medium. Subsequently, 1 mL of the enriched bacterial solution and 10 mM COBE were added to the restriction-screening liquid medium (30 mL). The culture was incubated at 25 °C and 180 rpm for 2–5 days until it became turbid. Subsequently, turbid restrictive liquid cultures were inoculated onto solid medium for single colony culture. The resulting single colonies were subjected to incubation and biotransformation experiments. The strains that exhibited the highest efficiency in transforming COBE into (*R*)-CHBE were finally screened, and the strains were conserved.

Total DNA was extracted from the best strains, and then the sequence was amplified as bacterial 16S rDNA using universal primers (upstream primer 5′ AGTTTGATCMTGGCTCAG 3′, downstream primer 5′ GGTTACCTTGTTACGACTT 3′), and the results were used for strain identification. The PCR products were subjected to 0.9% agarose gel electrophoresis, and then sequenced by Beijing Prime Biotechnology Co., Ltd. (Beijing, China)

### 3.3. Asymmetric Bioreduction Process

The best strains of wet cells were obtained and resuspended in a test tube containing 50 mL of pH 7.0 phosphate buffer and 0.1 g/mL glucose as a co-substrate. COBE was pre-dissolved in 5% (*v*/*v*) isopropanol, and the buffer was subsequently added to 5 mL. The reaction was incubated at 25 °C and 180 rpm for 30 h, centrifuged at 9000 rpm for 10 min, and the supernatant was extracted with an equal volume of ethyl acetate three times, and dried over anhydrous sodium sulfate. The obtained reduction product was dried until free of liquid, followed by successive addition of 10 μL acetic anhydride and 10 μL pyridine. The mixture was then boiled for 30 min. Finally, the resolved chiral product was dissolved in an appropriate amount of ethyl acetate. The sample was redissolved in 300 μL of ethyl acetate and analyzed by GC.

### 3.4. Analytical Methods

A gas chromatography Agilent CP7502 J&W CP ChirasilDex CB fitted with a chiral column (Machery Nagel; 25 m × 0.25 mm × 0.25 mm) was employed for the detection process. The inlet temperature was 250 °C, the column temperature was 110 °C, and the detector temperature was 250 °C. A hydrogen ion flame detector was employed with a split ratio of 1:15 and a flow rate of 1 mL/min. The retention time for COBE was 9 min, while those for (*R*)-CHBE and (*S*)-CHBE were 19 min and 19.8 min, respectively. Equations (1) and (2) evaluated the yield (X) and enantiomeric excess of *R*-CHBE (eep).

(1) $$X(\%)=\frac{p\times Ms}{q\times Mp}\times 100\%$$

Ms and Mp are the molecular weights of the substrate and the product, respectively. p and q represent the mass of the product at the end of the reaction and the initial mass of the substrate, respectively.

(2) $$eep=\frac{C_{R}-C_{s}}{C_{R}+C_{s}}\times 100\%$$

C_R_ and C_S_ represent the concentrations of *R*-CHBE and *S*-CHBE, respectively.

### 3.5. Selection of the Most Suitable Culture Medium

Screening for the optimal medium was conducted by studying the effect of the type of medium on the catalytic activity of the bacterium. LB medium (tryptone: 10 g/L; yeast extract: 5 g/L; sodium chloride: 10 g/L), modified LB medium (tryptone: 10 g/L; yeast extract: 10 g/L; sodium chloride: 10 g/L), TSB medium (tryptone: 17 g/L, soya peptone: 3.0 g/L, sodium chloride: 5 g/L, glucose: 2.5 g/L, chlorodimethyl phosphate: 2.5 g/L), and complete medium (glucose: 25 g/L, yeast extract: 3 g/L, ammonium sulphate: 5 g/L, magnesium sulphate: 0.25 g/L, dipotassium hydrogen phosphate trihydrate: 1.5 g/L, potassium dihydrogen phosphate: 1.5 g/L) were used for the fermentation of the engineered bacteria. The rest of the procedure is described in Section 3.3.

### 3.6. Effect of Different NADESs on Biotransformation of COBE

On the basis of the base medium screened in Section 3.5, the seed solution was inoculated into 150 mL of liquid fermentation medium at an inoculation rate of 3%. At the same time, 10 NADESs were added at 0.5% (*w*/*v*) of the fermentation broth (150 mL), respectively. The best strain wet cells (240 g/L) obtained from the co-culture were resuspended in test tubes with 0.1 M phosphate buffer, pH 7.0, and a final concentration of 40 mM COBE was added. The remainder of the procedure was carried out as in Section 3.3.

### 3.7. Effect of Secondary Addition of NADESs on the Biotransformation of COBE

The wet cells of the best strain (240 g/L) obtained by co-cultivation were resuspended in test tubes with 0.1 M phosphate buffer, pH 7.0, and 10 NADES were added again. The amount of NADES added was 0.5% (*w*/*v*) of the reaction system (5 mL), and the final concentration of COBE added was 40 mM. The rest of the procedure was the same as in Section 3.6.

### 3.8. Optimization of Biotransformation Conditions

Depending on the preferred NADES, adjust the addition gradient from 0.1% to 2% (*w*/*v*) according to the preferred addition method and proceed as in Section 3.6.

The best strain of wet cells enriched under NADES co-culture conditions was subjected to the reduction reaction at a temperature of 25–45 °C, a reaction time of 12–60 h, and buffer pH 5–10. At the same time, the best strain of wet cells without co-culture treatment was subjected to reduction reactions under the different parameters mentioned above. The rest of the procedure was the same as in Section 3.6.

### 3.9. Comparison of Assisted Catalysis of Related Components in Preferred NADES by Two Addition Methods

Addition method I: During the fermentation period, the best strain cells were co-cultured with the preferred NADES, single-component (choline chloride/urea) and two-component simple co-mixtures (choline chloride and urea) of the preferred NADES, respectively. The above components were added at 0.5% (*w*/*v*) of the fermentation broth (150 mL), respectively. The rest of the procedure was the same as in Section 3.6.

Addition method II: The best strain wet cells under co-culture conditions were collected, and then the preferred NADES, single-component (choline chloride/urea) and two-component simple co-mixtures (choline chloride and urea) of the preferred NADES, were added again in reaction buffer. The above components were added again at 0.5% (*w*/*v*) of the reaction system (5 mL). The rest of the procedure was performed as in Section 3.7.

### 3.10. Characterization of Cell Permeability Under Different Addition Conditions of NADES

The seed liquid of the best strain was inoculated into the fermentation broth (150 mL) at an inoculum rate of 3%. Meanwhile, three portions of the inoculated fermentation broths were added with 0.5% (*w*/*v*) of the preferred NADES: single component I (choline chloride), single component II (urea), and two-component simple co-mixtures (choline chloride and urea). The last portion of the fermentation broth was used as a blank control (only the seed liquid was added). After 24 h of incubation at 25 °C, 180 rpm, the appropriate amount of supernatant was obtained by centrifugation at 9000 rpm for 10 min, and then the OD260 and OD280 indexes were measured under UV. The bacterial precipitates were further re-collected in 5 mL of reaction buffer, and the above components were added separately. The supernatants were then collected under the same incubation conditions for testing.

### 3.11. FTIR Characterization of ChCl:U (1:2) and Its Single Components

The FTIR (Vertex 70, Bruker Corporation, Berlin, Germany) spectra of individual components of the preferred NADES were analyzed for spectral variations in the 4000–400 cm^−1^ wavelength region. The preferred NADES and its single component were structurally analyzed.

### 3.12. FCM Characterization of Cells Obtained by Different Treatment Conditions

An appropriate amount of fermentation broth without NADES treatment, with NADES co-culture treatment for 24 h, and with NADES treatment in buffer for an additional 24 h was taken. After centrifugation at 9000 rpm for 10 min, the supernatant was discarded and washed twice with 0.1 M pH 7.0 PB buffer, then resuspended in 1 mL of 0.1 M pH 7.0 phosphate buffer. Under light avoidance conditions, 200 μL of diluted propidium iodide (PI) staining solution was gently aspirated or beaten into the above resuspended bacterial solution, and then flow cytometry single stain detection was performed.

### 3.13. SEM Observation of Cells Obtained Under Different Treatment Conditions

Strain CGMCC No:28566 was subjected to the following treatments: (1) control (untreated cells without co-cultivation); (2) co-cultivation with 0.5% (*w*/*v*) ChCl:U (1:2) for 24 h; and (3) additional supplementation with 0.5% (*w*/*v*) ChCl:U (1:2) in the reaction buffer, followed by continued cultivation for 24 h. Adequate amounts of wet cells from the best strain under each treatment condition were collected and slowly added to pre-cooled 2.5% glutaraldehyde solution, fixed overnight, and centrifuged to remove the fixative. The samples were then dehydrated with 70%, 80%, 90%, and 100% ethanol, respectively, lyophilized, and subjected to gold spraying treatment. The structure was observed and analyzed under the scanning electron microscope (SEM) (JSM-6360LV, JEOL Ltd., Tokyo, Japan).

## 4. Conclusions

This study investigated the catalytic reactions under different conditions based on co-culture-assisted catalysis of NADSE. The ten NADESs were co-cultured with the CGMCC NO:28566 strain in fermentation broth for 24 h. The wet cell obtained under these conditions was subjected to a reduction reaction. Concurrently, the NADES, which possesses the qualities of enhancing substrate solubility and stabilizing enzyme activity, was incorporated into the reduction reaction for a period of 24 h, during which time the process was facilitated by the NADES. The outcomes of the two methodologies for catalytic additions demonstrated that the enantiomeric excess values were largely unperturbed. The former approach, in which ChCl:U (1:2) [28,29,30] assisted catalysis was found to be the most effective, with a 58.2% increase in (*R*)-CHBE yield. However, in the latter case, the lowest yield of (*R*)-CHBE was found to have decreased from 54.2% to 29.6%. It is likely that the observed effects were due to the action of NADES on the bacterial cells during the co-culture stage, resulting in changes to their membrane permeability. The addition of NADES to this environment may have further increased the extent of these changes, potentially impairing cellular activity. Consequently, we have elected to employ the use of NADES during the enrichment phase of fermentation, with the intention of facilitating catalysis.

Furthermore, the objective of this investigation is to determine whether the observed effect of ChCl:U (1:2) can be attributed to the individual components present in the solvent or whether a simple blend of single components can achieve the same result. It was thus demonstrated that a single component or simple co-mixture exerted a certain degree of assisted catalysis effect. Furthermore, the auxiliary effect of simple co-mixture was found to be slightly higher than that of a single component. However, the effect was considerably less pronounced than that observed with ChCl:U (1:2) solvent assistance. The OD260 and OD280 values were determined, and the results demonstrated that the OD260 and OD280 values were indeed elevated in comparison to the control due to the single component or simple co-mixture. However, the elevation of the two values due to ChCl:U (1:2) was more significant. The trends observed in the OD260 and OD280 values were also corroborated by the results of the catalysis of the components.

The selected wild-type enzyme possesses an intrinsic catalytic advantage due to its native multi-enzyme synergy system, enabling superior substrate specificity compared to artificially engineered strains. Moreover, the wild-type strain demonstrates enhanced growth kinetics and operational stability, eliminating the need for genetic-instability-prone regulatory interventions required in engineered counterparts. The screened wild strain exhibits remarkable environmental tolerance and a broad substrate spectrum. These inherent enzymatic virtues establish a robust biological foundation for the NADES system investigated herein, while providing a diverse enzymatic repertoire for discovering pivotal biocatalysts.

## Figures and Tables

**Table 1:** NADES for experiments.

Component Ⅰ	Component Ⅱ	Proportion (mol)
betaine	glycerol	1:2
betaine	xylitol	1:2
betaine	urea	1:2
choline chloride	urea	1:2
choline chloride	xylitol	1:2
choline chloride	glucose	3:2
choline chloride	glycerol	1:2
choline chloride	lysine	1:2
choline chloride	glutathione	1:1
proline	urea	1:2

**Table 2:** Quantitative analysis of the OD260 and OD280 values following the application of various treatments to ChCl:U (1:2) and its components.

Groups	Net OD260 nm	Net OD280 nm
Control	0.122 ± 0.015	0.158 ± 0.010
Urea * 24 h	0.135 ± 0.002	0.166 ± 0.012
Choline chloride * 24 h	0.145 ± 0.019	0.171 ± 0.011
Urea + Choline chloride *24 h	0.173 ± 0.023	0.191 ± 0.009
ChCl:U (1:2) * 24 h	0.351 ± 0.022	0.385 ± 0.024
Urea # 24 h	0.175 ± 0.016	0.193 ± 0.007
Choline chloride # 24 h	0.183 ± 0.013	0.199 ± 0.020
Urea + Choline chloride # 24 h	0.211 ± 0.021	0.341 ± 0.005
ChCl:U (1:2) # 24 h	0.583 ± 0.007	0.671 ± 0.014

Note: * represents co-culture; # represents re-addition in reaction buffer.

## Data Availability

All data generated or analyzed during this study are included in this article.
